# Supplementary material for: Prevalence and Antibiotic Resistance in Campylobacter spp. Isolated from Humans and Food-Producing Animals in West Africa: A Systematic Review and Meta-Analysis
Source: Pathogens. 2022 Jan 24;11(2):140. doi: 10.3390/pathogens11020140 (PMC8877155; doi:10.3390/pathogens11020140)
Supplement: Supplementary file 1 [file pathogens-11-00140-s001.zip › pathogens-1521673 supplement_EKP.pdf]

Prevalence and antibiotic resistance profiles of *Campylobacter* spp. isolated from humans and animals in West Africa: a systematic review and meta-analysis

---

Search Strategy

Conducted on 31 July 2021

Database: Medline

#	Searches	Results
#1	Campylobacteriosis[Mesh] OR Campylobacter[Mesh] OR campylob*[tw]	19,249
#2	West Africa[Mesh] **OR** Benin[tw] OR Burkina Faso[tw] OR Cape Verde[tw] OR Côte d'Ivoire[tw] OR Gambia[tw] OR Ghana[tw] OR (Guinea[tw] NOT guinea pig*[tw]) OR Guinea-Bissau[tw] OR Liberia[tw] OR Mali[tw] OR Mauritania[tw] OR Niger[tw] OR Nigeria[tw] OR Saint Helena[tw] OR Senegal[tw] OR Sierra Leone[tw] OR Togo[tw] OR Western Africa*[tw] OR West Africa*[tw] AND "English"[language]	104,295
#3	#1 AND #2	**145**

* instruct the database to search for variations in spelling and variant endings of the root word "campylob".

Database: Directory of Open Access Journals (DOAJ)

#	Searches	Results
**1**	Campylobact* AND Benin	4
**2**	Campylobact* AND Burkina Faso	1
**3**	Campylobact* AND Cape Verde	0
**4**	Campylobact* AND Côte d'Ivoire	1
**5**	Campylobact* AND Gambia	3
**6**	Campylobact* AND Ghana	9
**7**	Campylobact* AND Guinea NOT guinea pig*	0
**8**	Campylobact* AND Guinea-Bissau	1
**9**	Campylobact* AND Liberia	0
**10**	Campylobact* AND Mali	1
**11**	Campylobact* AND Mauritania	0
**12**	Campylobact* AND Niger	3
**13**	Campylobact* AND Nigeria	15
**14**	Campylobact* AND Saint Helena	0

**15**	Campylobact* AND Senegal	4
**16**	Campylobact* AND B Sierra Leone	0
**17**	Campylobact* AND Togo	1
**18**	Campylobact* AND Western Africa	4
**19**	Campylobact* AND West Africa	10
	**Total**	**57**

*instruct the database to search for variations in spelling and variant endings of root words".

Database: Google Scholar

No	Searches	Results
**1**	allintitle: Campylobacter OR campylobacteriosis AND Benin	2
**2**	allintitle: Campylobacter OR campylobacteriosis AND Burkina Faso	6
**3**	allintitle: Campylobacter OR campylobacteriosis AND Cape Verde	0
**4**	allintitle: Campylobacter OR campylobacteriosis AND Côte d'Ivoire	4
**5**	allintitle: Campylobacter OR campylobacteriosis AND Gambia	1
**6**	allintitle: Campylobacter OR campylobacteriosis AND Ghana	6
**7**	allintitle: Guinea Campylobacter OR Campylobacteriosis -"guinea pig"	7
**8**	allintitle: Campylobacter OR campylobacteriosis AND Guinea-Bissau	0
**9**	allintitle: Campylobacter OR campylobacteriosis AND Liberia	0
**10**	allintitle: Campylobacter OR campylobacteriosis AND Mali	0
**11**	allintitle: Campylobacter OR campylobacteriosis AND Mauritania	0
**12**	allintitle: Campylobacter OR campylobacteriosis AND Niger	0
**13**	allintitle: Campylobacter OR campylobacteriosis AND Nigeria	71
**14**	allintitle: Campylobacter OR campylobacteriosis AND Saint Helena	0
**15**	allintitle: Campylobacter OR campylobacteriosis AND Senegal	13
**16**	allintitle: Campylobacter OR campylobacteriosis AND Sierra Leone	0
**17**	allintitle: Campylobacter OR campylobacteriosis AND Togo	0
**18**	allintitle: Campylobacter OR campylobacteriosis AND Western Africa	0
**19**	allintitle: Campylobacter OR campylobacteriosis AND West Africa	5
	**Total**	**128**

Database: African Index Medicus

#	Searches	Results
**1**	Campylobacter	44
**2**	Campylobacteriosis	3
**3**	**1 and 2 (Restrict to English)**	**34**

Database: African Journals Online (AJOL)

#	Searches	Results
**1**	campylob*	**427**

*instruct the database to search for variations in spelling and variant endings of the root word "campylob".
